# Adaptive Evolution of Industrial *Lactococcus lactis* Under Cell Envelope Stress Provides Phenotypic Diversity

**DOI:** 10.3389/fmicb.2018.02654

**Published:** 2018-11-05

**Authors:** María Jesús López-González, Susana Escobedo, Ana Rodríguez, A. Rute Neves, Thomas Janzen, Beatriz Martínez

**Affiliations:** ^1^DairySafe Group, Instituto de Productos Lácteos de Asturias (IPLA)-Consejo Superior de Investigaciones Científicas (CSIC), Villaviciosa, Spain; ^2^Chr. Hansen A/S, Hørsholm, Denmark

**Keywords:** dairy starter, bacteriocin, stress, adaptive evolution, cell wall

## Abstract

*Lactococcus lactis* is widely used as a starter in the manufacture of cheese and fermented milk. Its main role is the production of lactic acid, but also contributes to the sensory attributes of cheese. Unfortunately, the diversity of suitable strains to be commercialized as dairy starters is limited. In this work, we have applied adaptive evolution under cell envelope stress (AE-CES) as means to provide evolved *L. lactis* strains with distinct physiological and metabolic traits. A total of seven strains, three of industrial origin and four wild nisin Z-producing *L. lactis*, were exposed to subinhibitory concentrations of Lcn972, a bacteriocin that triggers the cell envelope stress response in *L. lactis.* Stable Lcn972 resistant (Lcn972R) mutants were obtained from all of them and two mutants per strain were further characterized. Minimal inhibitory Lcn972 concentrations increased from 4- to 32-fold compared to their parental strains and the Lcn972R mutants retained similar growth parameters in broth. All the mutants acidified milk to a pH below 5.3 with the exception of one that lost the lactose plasmid during adaptation and was unable to grow in milk, and two others with slower acidification rates in milk. While in general phage susceptibility was unaltered, six mutants derived from three nisin Z producers became more sensitive to phage attack. Loss of a putative plasmid-encoded anti-phage mechanism appeared to be the reason for phage susceptibility. Otherwise, nisin production in milk was not compromised. Different inter- and intra-strain-dependent phenotypes were observed encompassing changes in cell surface hydrophobicity and in their autolytic profile with Lcn972R mutants being, generally, less autolytic. Resistance to other antimicrobials revealed cross-protection mainly to cell wall-active antimicrobials such as lysozyme, bacitracin, and vancomycin. Finally, distinct and shared non-synonymous mutations were detected in the draft genome of the Lcn972R mutants. Depending on the parental strain, mutations were found in genes involved in stress response, detoxification modules, cell envelope biogenesis and/or nucleotide metabolism. As a whole, the results emphasize the different strategies by which each strain becomes resistant to Lcn972 and supports the feasibility of AE-CES as a novel platform to introduce diversity within industrial *L. lactis* dairy starters.

## Introduction

Dairy starters have been applied for the production of fermented dairy products more than a century ago, when the first dairy starter strains were isolated and intentionally added to milk. The main components of dairy starters are lactic acid bacteria (LAB) and, in particular, *Lactococcus lactis* is the most common acidifying strain used in the production of cheese. According to their composition, cheese starters are classified into undefined and defined starters. Undefined starters are complex mixtures of unknown composition, whereas defined starters are blends of well-characterized strains of one (single) or multiple (mixed) species (Rodríguez et al., 2012). These starter strains have been isolated and selected according to their technological properties, namely based on fast growth and acidification rate in milk, proteolytic activity and bacteriophage resistance. Other features such as the synthesis of aroma compounds, texturing agents and inhibitory compounds are also of interest (Derkx et al., 2014). Commercial defined starters are currently available in highly concentrated frozen and freeze-dried formats, ready to be added directly to vat milk to minimize the risk of starter contamination during handling and changes in starter composition in the factory. Overall, the use of starters results in reliable cheese quality and, most importantly, in a more consistent acidification rate that allows cheese making be conducted on a fixed time schedule (Johnson, 2017).

Changes in consumer preferences toward less additives and artificial ingredients in fermented products put pressure on companies engaged in the production of starters to expand their strain portfolio to satisfy these demands (Johansen et al., 2015). However, there is a general consensus that the biodiversity of available commercial starters is relatively small to develop defined starter blends for novel applications. Consequently, either new sources or novel strategies for strain development are required (McAuliffe, 2018). Starter improvement may be approached by knowledge-based screenings of large culture collections, looking for the desired combination of properties, or by evolving new phenotypes from existing starters. The former approach is limited by the fact that not all strains survive the conditions imposed by industrial production, whereas the latter may be applied to strains whose production at large-scale has been already optimized. The success of strain improvement based on natural selection and experimental evolution has been recently reviewed by Johansen (2018). Besides classical mutagenesis and standard or cutting-edge genome editing technologies, knowledge-based strategies to diversify may include positive selection under a particular condition. For example, to increase yogurt sweetness, growth of *Streptococcus thermophilus* mutants able to consume the galactose moiety of lactose and excrete the glucose moiety could be isolated. First, growth on galactose to select for galactose positive mutants was approached, followed by subsequent selection on the non-metabolized glucose analog 2-deoxyglucose (Sørensen et al., 2016). Additionally, adaptive evolution experiments that involved serial propagation under selective conditions have also been applied to LAB to evolve strains with specific phenotypes (see Bachmann et al., 2017 for a recent review). Examples of adaptive evolution in *L. lactis* include adaptation to high temperature (Chen et al., 2015), or adaptation of a plant isolate to grow in milk (Bachmann et al., 2012).

An alternative route to diversify *L. lactis* populations may be the use of bacteriocins in evolution experiments. Bacteriocins are bacterial ribosomally synthesized antimicrobial peptides. Those produced by LAB have received much attention due to their biotechnological potential as food biopreservatives, based on their potent inhibitory activity against foodborne pathogens and spoilage microorganisms. LAB bacteriocins encompass a large and diverse group of peptides with multiple structures that kill target bacteria by interfering with cell envelope functions, either by disruption of membrane permeability by pore formation and/or inhibition of cell wall synthesis (Álvarez-Sieiro et al., 2016). It has also been shown that bacteriocin resistant mutants (BacR) can be isolated under laboratory conditions. BacR mutants may display altered phenotypes from changes in cell wall constituents and membrane fluidity to alterations of carbon metabolism. The latter occurs namely by mutation or repression of bacteriocin receptors which are also involved in sugar uptake (Tessema et al., 2011; Bastos et al., 2015). However, most of these studies have focused on mutants of main foodborne pathogens such as *Listeria monocytogenes* or *Staphylococcus aureus*. Much less is known on the impact of bacteriocin resistance in industrially relevant bacteria such as *L. lactis*. Nisin resistance in *L. lactis* IL1403 has been linked to an increased D-alanylation of lipoteichoic acids and a thickened cell wall at the septum (Kramer et al., 2008). Transcriptomic studies further suggested that other mechanisms (e.g., ABC transporters and metabolic adaptations) may be also involved (Kramer et al., 2006). The phenotypic consequences of resistance to the lantibiotic lacticin 3147 have also been characterized in *L. lactis* IL1403 (Guinane et al., 2006). Resistance to other anti-lactococcal bacteriocins such as lactococcin G and LsbB has been associated with mutations of the enzyme undecaprenyl pyrophosphate phosphatase (UppP), involved in cell wall biosynthesis, and of a membrane metallopeptidase (YvjB), respectively (Kjos et al., 2014; Miljkovic et al., 2016). These proteins are suggested to be bacteriocin receptors required for antimicrobial activity.

Lactococcin 972 (Lcn972) is a non-pore forming bacteriocin only active against *Lactococcus* that inhibits cell wall biosynthesis at the septum by specifically binding to lipid II (Martínez et al., 2008). In line with this mode of action, Lcn972 triggers the cell envelope stress response in *Lactococcus* through the activation of the two component system CesSR (Martínez et al., 2007). Although we were not able to isolate spontaneous mutants after a single exposure to Lcn972, a stable resistant mutant of the laboratory strain *L. lactis* MG1614 could be selected by subculturing in the presence of increasing Lcn972 concentrations. The characterization of a single resistant mutant demonstrated that changes on the cell surface, along with chromosomal deletions and transcriptional gene activation mediated by insertion sequences, had occurred (Roces et al., 2012a,b). This mutant revealed cross-resistance to lysozyme and nisin and insensitivity to the bacteriophage c2 (Roces et al., 2012a). More recently, to assess if industrial strains are also prone to Lcn972 plasticity, we have applied the same procedure to the cheese starter *L. lactis* IPLA947. *L. lactis* R5, a Lcn972 resistant mutant (Lcn972R) four times more resistant than *L. lactis* IPLA947, showed increased resistance to oxidative stress without compromising the acidification rate in milk (López-González et al., 2018).

These previous results encourage us to propose that Adaptive Evolution under Cell Envelope Stress (AE-CES), using Lcn972 as a stressor, might be an option to diversify industrial *L. lactis*, i.e., to introduce new phenotypes in existing dairy starters. Thus, the aim of this work was to apply AE-CES to seven *L. lactis* strains of different origins (industrial and raw milk cheese isolates), and assess the phenotypes of their Lcn972R mutants with special emphasis in milk growth, surface properties and stress survival. Both unique and common phenotypes were observed. Moreover, distinct point mutations could be detected in the draft genome sequence of thirteen Lcn972R mutants that revealed the heterogeneity of the different strategies of *L. lactis* to cope with the stress imposed by Lcn972.

## Materials and Methods

### Bacterial Strains and Growth Conditions

*Lactococcus lactis* strains used in this work and their sources and properties are shown in Table 1. Additionally, *L. lactis* IPLA947 (Cárcoba et al., 2000), the acidifying strain of the *Afuega′l Pitu* cheese starter, and its Lcn972R mutant R5 (López-González et al., 2018) were also included for the phenotypic and genotypic tests. *L. lactis* was routinely grown at 30°C statically in LM17 (Biokar Diagnostics) that incorporates lactose at 0.5% (w/v) in its composition. For testing lactose fermentation, a basal broth was prepared with: (w/v) 0.5% tryptone (Oxoid), 0.3% meat extract (Biokar Diagnostics), and bromocresol purple dye (BCP) at 0.004% supplemented with either lactose (BCP-lac) or glucose (BCP-glc) at 0.5%. *L. lactis* MG1614 was grown in M17 (Oxoid) supplemented with glucose (GM17) at 0.5% and used as indicator for Lcn972 quantification. *Micrococcus luteus* NCIMB8166 was used as indicator for nisin quantification and was grown in Tryptic Soy broth (TSB, Biokar Diagnostics) at 37°C. Plates were prepared with agar at 2%. Frozen stocks were kept at -80°C in the presence of 10% glycerol. Working culture stocks were prepared from overnight (16–18 h) cultures started with a single colony. Glycerol was added to a final concentration of 10% and 40 μl-aliquots were stored at -80°C. Before each experiment, one aliquot was unfrozen and used to inoculate 4 ml of LM17. Growth curves were carried out in a Benchmark Plus Microplate spectrophotometer (Bio-Rad Laboratories). Growth was started by diluting overnight cultures in pre-warmed LM17 to an optical density at 600 nm (OD_600_) of 0.05. Growth rate (μ) was calculated by linear regression of ln(OD_600_) versus time in the exponential phase in, at least, two independent experiments.

**Table 1:** Source and properties of *Lactococcus lactis* subsp. *lactis* strains used in this work.

Strain-code	Source and properties	Reference	Lcn972 MIC^1^ (AU/ml)	Growth rate^2^ (h^-1^)
*L. lactis* L81	Commercial mesophilic starter. Acidifying strain	CHCC collection	10	0.69 ± 0.10
L81-D1	Resistant to Lcn972	This work	80	0.83 ± 0.04^∗^
L81-E2	Resistant to Lcn972	This work	320	0.79 ± 0.01
*L. lactis* L62	Commercial mesophilic starter. Acidifying strain	CHCC collection	10	0.84 ± 0.01
L62-C9	Resistant to Lcn972	This work	320	0.89 ± 0.04
L62-G9	Resistant to Lcn972	This work	80	0.80 ± 0.02
*L. lactis* L98	Bioprotective culture. Nisin A producer	CHCC collection	40	0.95 ± 0.01
L98-C1	Resistant to Lcn972	This work	160	0.90 ± 0.04^∗^
L98-E2	Resistant to Lcn972	This work	160	0.81 ± 0.09^∗^
*L. lactis* IPLA517	Raw milk cheese, nisin Z producer	Martínez et al., 1995	10	0.95 ± 0.01
517-B5	Resistant to Lcn972	This work	320	0.74 ± 0.06
517-C6	Resistant to Lcn972	This work	160	0.82 ± 0.16
*L. lactis* IPLA641	Raw milk cheese, nisin Z producer	Martínez et al., 1995	10	0.98 ± 0.09
641-C8	Resistant to Lcn972	This work	80	0.87 ± 0.09
641-D8	Resistant to Lcn972	This work	160	0.79 ± 0.01
*L. lactis* IPLA729	Raw milk cheese, nisin Z producer	Martínez et al., 1995	10	1.08 ± 0.04
729-D10	Resistant to Lcn972	This work	160	0.97 ± 0.00
729-F9	Resistant to Lcn972	This work	160	0.90 ± 0.03^∗^
*L. lactis* IPLA1064	Raw milk cheese, nisin Z producer	Martínez et al., 1995	10	0.97 ± 0.00
1064-C11	Resistant to Lcn972	This work	160	0.81 ± 0.01
1064-E11	Resistant to Lcn972	This work	160	0.78


*^*1*^MIC, Minimal Inhibitory Concentration. AU, Arbitrary Units.*

*^*2*^Growth rate in LM17 broth at 30°C. Results are the mean ± standard deviation (n = 2).*

*^∗^Statistically different (p < 0.05) compared to the equivalent wild-type strain.*

### Minimal Inhibitory Concentration

Susceptibility to Lcn972 was determined by the broth microdilution method as previously described (Martínez et al., 2008). Purified Lcn972 stock was prepared in 50 mM sodium phosphate buffer, pH 6.8, with a specific activity of 34.6 AU/μg (12,800 AU/ml, 370 μg/ml) and was kept at -20°C. Twofold dilutions (100 μl) were made in broth and placed on microtiter plates. Wells were inoculated with 100 μl of an exponentially growing culture adjusted to OD_600_ of 0.05 and further diluted 1:100. Plates were incubated for 24 h at 30°C.

### Adaptive Evolution Experiments

**FIGURE 1:**
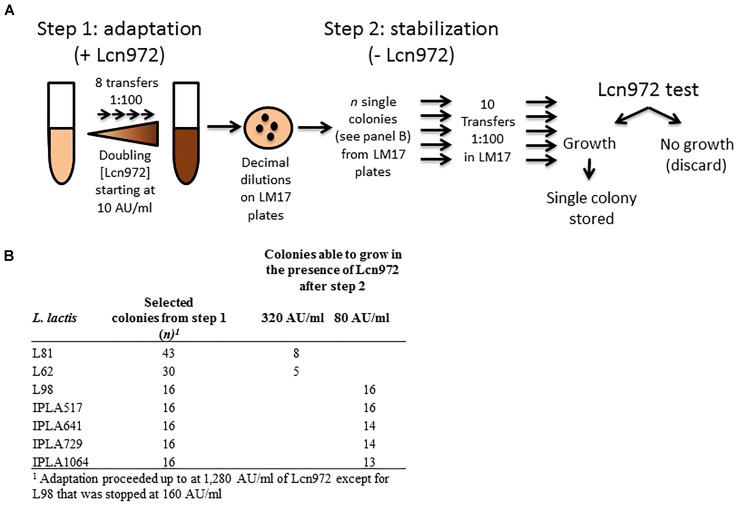
Outline of the adaptive evolution experiment to isolate Lcn972R mutants. **(A)** During the adaptation step, *Lactococcus lactis* strains were subcultured in LM17 with increasing (twofold) Lcn972 concentrations. After this step, several colonies from the adapted cultures grown at the highest Lcn972 concentration were subcultured in the absence of Lcn972 (stabilization step). **(B)** Number of colonies subjected to the stabilization step and number of those which still retained resistance to Lcn972 after stabilization.

Adaptation to Lcn972 proceeded essentially as described by Roces et al. (2012a) and is depicted in Figure 1A. Cultures were started with a single colony in LM17. After overnight incubation at 30°C, they were used to inoculate at 1% (v/v) 10 ml of pre-warmed LM17 containing 10 AU/ml of Lcn972. After incubation at 30°C for 16 h, the same procedure was applied, doubling the concentration of Lcn972 in each round. A total of eight consecutive transfers were done into fresh LM17, starting at 10 AU/ml up to 1,280 AU/ml, with the exception of L98 that was stopped after 5 transfers at 160 AU/ml. Decimal dilutions of each culture grown at the highest Lcn972 concentration were spread on LM17 plates without Lcn972 to isolate single colonies. From *L. lactis* L81 and L62, 43, and 30 colonies were picked, respectively. For nisin producers, 16 colonies per strain were picked (Figure 1B). Each colony was transferred to 1-ml deepwell microtiter plates filled with LM17 and grew for 24 h at 30°C. These microtiter plates were replicated by inoculating at 1% (v/v) new LM17 deepwell plates that were incubated at 30°C for 24 h. After 10 serial transfers, the susceptibility to Lcn972 was checked. To do so, cells from each well were diluted 1:100 in the case of *L. lactis* L81 and L62 or 1:160 for nisin producers in fresh LM17 and 5 μl were used to inoculate 200 μl of LM17 plus Lcn972 at 320 AU/ml (L81 and L62) or 80 AU/ml (nisin producers). Two cultures per *L. lactis* strain able to grow in the presence of Lcn972 were streaked on a LM17 plate and a single colony was stored at -80°C.

### Milk Acidification and Production of Lactic Acid

Skim milk powder (Difco) was reconstituted at 11% (w/v) with distilled water and treated at 100°C for 30 min. Overnight LM17 cultures were centrifuged and washed once with Ringer solution (Merck, Germany). Milk (10 ml) was inoculated at 3% (v/v) with the cell suspension and incubated at 30°C for 6 h. pH was measured with the pHmeter micropH 2001 (Crison, Spain). Acidification curves of L81 and L62 and their Lcn972 resistant mutants (Lcn972R) were started by inoculation of 40 ml of milk with the cell suspension at 2% (v/v) and pH was followed during the incubation at 30°C with the real-time pHmeter Orion^TM^ Versa^TM^ Start (Thermo Scientific Inc., United States) every 30 min for 20 h. Maximum acidification rate (*V*_m_) and the time at which pH 4.6 was reached (Te) were used as acidification parameters as described by Kristo et al. (2003).

Lactic acid production was analyzed by HPLC using an ICSep-ICE-ION-300 column with 0.0085 N H_2_SO_4_ as mobile phase at flow rate of 0.4 ml/min and operating temperature of 65°C. Lactic acid was detected at 210 nm by a 996 photodiode array detector (Waters). Milk samples (1 ml) were deproteinized with 5 ml of 4.5 mM H_2_SO_4_ for 1 h at 37°C in a rotary shaker. After centrifugation (16,000 × *g*, 15 min, 4°C), supernatants were filtered through a 0.45 μm PTFE filter (VWR international). All the experiments were carried out with at least two independent cultures.

### Nisin Production

Nisin was quantified by the agar well diffusion method. Plates were prepared by inoculating melted TSB agar 1.2% with 10^5^ colony forming unit (CFU) per ml of *M. luteus* NCIMB8166. After solidification, wells (4 mm diameter) were made and filled with 20 μl of culture or milk supernatants obtained by centrifugation (15,400 × *g*, 15 min, 4°C). Plates were incubated at 37°C for 24 h to score inhibition halos. A calibration curve was prepared with pure nisin (a gift from Applin & Barret, United Kingdom) with concentrations from 5 to 25 μg/ml diluted in 0.05% acetic acid. Quantification was performed in two independent cultures.

### Bacteriophage Susceptibility

Different phages from the Chr. Hansen collection infecting *L. lactis* L81, L62, and L98 were tested by the double layer assay. Phage decimal dilutions prepared in LM17 were spotted (10 μl) onto LM17 plates containing 10 mM Ca^++^ and overlaid with soft LM17 agar (0.7%) inoculated with 100 μl of the *L. lactis* overnight culture. Clear halos and isolated lysis plaques were observed after overnight incubation at 30°C. When required, plaque forming units per ml (PFU/ml) were estimated after incubating 100 μl of the phage dilution with 100 μl of the bacterial culture for 10 min, then mixed with 8 ml of soft LM17 and poured on a LM17 plate. To identify phages infecting the *L. lactis* nisin producers IPLA517, IPLA641, IPLA729, and IPLA1064, a growth inhibition test in milk was carried out. Bacterial cultures were challenged with 62 phages at *ca*. 10^6^ PFU/ml and the pH was monitored. When the pH of a control culture without added phage was at 5.0, the pH of the infected culture was recorded, and a phage was considered infective when the pH difference was more than 0.5.

### Surface Properties and Autolysis

Cell surface hydrophobicity was measured by the Microbial-Adhesion To Solvent (MATS) protocol using hexadecane and stationary phase cells as described by Roces et al. (2012a). The degree of autolysis was determined according to Meyrand et al. (2007). Briefly, exponentially growing cells (OD_600_ = 0.3–0.5) were harvested, washed with 50 mM potassium phosphate buffer, pH 7.0, and resuspended in the same buffer supplemented with Triton X-100 at 0.05%. The OD_600_ of 300 μl-aliquots of the cell suspension was followed in the microtiter reader for 6 h at 30°C. Three biological replicates per strain were assayed. Cell suspensions without Triton X-100 were used as controls. The OD_600_ values were expressed as % of the initial OD_600_. For comparison, values of %OD_600_ after 150 min of incubation were taken.

### Survival to Heat and Acidic pH

Cells from overnight cultures were harvested by centrifugation (15,400 × *g*, 5 min). For the heat shock, cells from 200 μl-aliquots were washed with Ringer solution and diluted in 1 ml of the same solution (approximately 4–5 × 10^8^ CFU/ml). Cell suspensions were divided into two 0.5 ml-aliquots, one was hold in a thermoblock (VWR international) at 50°C for 30 min and the other left at room temperature. All the tubes were transferred to ice and decimal dilutions immediately prepared in Ringer solution. CFU/ml counts were estimated after spotting (6×) 5 μl of each dilution on LM17 plates. The experiment was carried out with three biological replicates. For the acidic shock, cells from 1 ml were washed twice with 150 mM NaCl, and finally resuspended in 0.5 ml of 150 mM NaCl. Each cell suspension (100 μl) was diluted in 900 μl of 150 mM NaCl and in 150 mM NaCl adjusted to pH 2 with HCl. Samples were prepared in duplicate and incubated for 30 min at 30°C. After the incubation, the tubes were centrifuged and the pellet resuspended in 1 ml of phosphate buffered saline (PBS, 10 mM Na_2_HPO_4_, 1.8 mM KH_2_PO_4_, 137 mM NaCl, 2.7 mM KCl, pH 7.4). Decimal dilutions were prepared in PBS to neutralize the pH and CFU/ml calculated as described after the heat shock. Survival was determined as log(Nt/No) where Nt and No are CFU/ml of treated samples and non-treated controls, respectively. In both experiments, three independent cultures per strain were used.

### Resistance to Antimicrobials

Decimal dilutions of exponentially growing cultures (OD_600_ = 0.3–0.5) were prepared in Ringer solution and spotted (5 μl) on LM17 plates supplemented with each antimicrobial at final concentrations of 1 μg/ml bacitracin, 0.1 mg/ml penicillin G, 0.4 μg/ml vancomycin and 0.5 mg/ml lysozyme from chicken egg white (all purchased from Sigma). LM17 plates supplemented with 5% NaCl were also prepared. Resistance to nisin (5 μg/ml) was also checked for all the starter strains and Lcn972R mutants but the nisin producers.

### Plasmid Isolation and Plasmid Curation

Plasmids were isolated from overnight cultures according to O’sullivan and Klaenhammer (1993). For curing experiments, *L. lactis* IPLA517 was grown in LM17 at 37°C for 24 h in the presence of novobiocin at 2 μg/ml. These cultures were transferred daily to LM17 with increasing novobiocin concentrations (5, 10, and 15 μg/ml). Cultures grown at 15 μg/ml of novobiocin were spread on LM17 plates to isolate single colonies.

### Identification of Non-synonymous Single Point Mutations

Draft genomes sequences were obtained at the sequencing facilities at Chr. Hansen. To perform full genome sequencing, DNA of the selected strains was isolated using the DNA DNeasy Blood and Tissue kit with the protocol for Gram-positive bacteria (Qiagen, Germany) and sequenced on the Illumina MiSeq platform with 2 × 250-bp paired-end sequencing (Illumina, United States). Sequencing reads were trimmed, analyzed, and assembled using CLC Genomics Workbench 10.1.1 (Invitrogen). The assembled contigs were annotated by RASTtk (Brettin et al., 2015). Whole Genome Shotgun projects have been deposited at DDBJ/ENA/GenBank under the bioproject accession number PRJNA492214. Detection of single point mutations in the genomes of the Lcn972R mutants was performed with CLC Genomics Workbench 10.1.1 (Invitrogen). Variants located in mobile elements and prophages were disregarded. Moreover, BLASTN^1^ was used to identify possible sequencing/assembling errors found in the wild type genome used as reference due to low coverage or low quality readings. Mutations found in plasmid-encoded genes which were also present in the chromosome (e.g., oligopeptide transport genes-*opp*) were not included in the analysis as the extra-copy may influence the outcome of the variant caller. Heatmap for representing the mutations found in the Lcn972R mutants was prepared with Heatmapper (Babicki et al., 2016). Hierarchical clustering was done by the average linkage method and distances were computed by the Spearman rank correlation. Functional analysis of the non-synonymous mutations detected in the Lcn972R strains was performed with GSEA_pro^2^ as implemented in Genome2D^3^. The SIFT algorithm was used to predict if an amino acid substitution affects protein function^4^.

### Statistical Analysis

Results are reported as mean ± SD where appropriate. Differences between the wild type strains and their Lcn972R derivatives were assessed by one-tailed *t-*test as implemented in Microsoft Excel 2010 (2010 Microsoft Corporation). *p* < 0.05 was considered to be significant.

## Results

### Adaptive Evolution Under Cell Envelope Stress (AE-CES) Applied to Industrial and Dairy Strains Selects for Stable *L. lactis* Mutants Resistant to Lcn972

To appreciate the value of AE-CES as means to evolve *L. lactis* and provide new phenotypes, this procedure was applied to seven wild type (WT) *L. lactis* subsp. *lactis* strains from different sources (Table 1). *L. lactis* L81 and L62 are used as acidifying strains in commercial starter blends. L98 is a nisin A producer, available as a bioprotective culture. The four nisin Z producers (IPLA517, IPLA641, IPLA729, and IPLA1064) were selected from the IPLA-CSIC laboratory collection. These strains were isolated from homemade raw milk cheeses made without starters in Northern Spain, and were chosen as representatives of each of the plasmid profiles found among the 23 isolates characterized by Martínez et al. (1995). They are genetically very closely related, according to their identical *Sma*I band pattern resolved by pulsed-field gel electrophoresis PFGE (our own unpublished results). Nonetheless, they were all subjected to AE-CES to determine if they generate similar outcomes.

The seven WT *L. lactis* strains were susceptible to Lcn972 with a MIC of 10 AU/ml and 40 AU/ml for L98 (Table 1). The AE-CES experiment involved two steps (see Figure 1A). In the first step, cultures were adapted to grow in increasing Lcn972 concentrations (adaptation step). All the cultures were able to grow up to 1,280 AU/ml (128× MIC) of Lcn972. The only exception was L98 that grew poorly at concentrations over 160 AU/ml (4× MIC). From each adapted culture, single colonies were picked and subjected to the stabilization step, consisting in ten serial transfers in LM17 without Lcn972. This step was carried out to enrich for the fittest clones. Then, their ability to grow in the presence of Lcn972 was tested Not all of them were able to grow (Figure 1B), suggesting that, in some cases, resistance to Lcn972 may be lost during the stabilization step. From two independent stabilized cultures per strain that retained the Lcn972R phenotype, a single colony was isolated, named after its parental strain, and stored for further characterization. Their Lcn972 MICs are shown in Table 1. MICs were increased from 4- to 32-fold. Most frequently, the MIC value was 160 AU/ml, while three mutants reached 320 AU/ml. It is important to note that the inoculum for the MIC determination differs from that used in the Lcn972 test. MIC plates were inoculated with approximately 1.5 × 10^5^ CFU/ml from exponentially growing cultures, while the Lcn972 test was carried out with stationary phase cultures and inoculated with 3.0 – 6.0 × 10^5^ CFU/ml. This difference in the inoculum may explain why Lcn972R mutants from *L. lactis* L81 and L62 had MICs below 320 AU/ml, the Lcn972 concentration used in the Lcn972 test. Moreover, the MICs were also below 1,280 AU/ml, the highest concentration used during adaptation. In this case, the transitory CesSR response must be activated during adaptation, which likely helps to cope with the stress and may allow growth at high Lcn972 concentrations.

In general, according to their growth rate in LM17 (Table 1), growth of the Lcn972R mutants was not hampered under laboratory conditions. *L. lactis* L81-D1 grew even faster than its parent (L81) (*p* < 0.05). On the contrary, *L. lactis* IPLA729-F9 and the two mutants from L98 were slower (Table 1). Furthermore, while the final OD_600_ in LM17 overnight cultures was similar to that of the parental strains (2.5–3.0) (data not shown), the mutants L98-C1 and L98-E2 reached a low OD_600_ compared to that of L98 (0.8 vs. 2.5, respectively). The bioprotective culture *L. lactis* L98 had a particular behavior. When streaked on LM17 plates, two colony phenotypes were always observed with an estimated frequency of 50%: a typical lactococcal white and smooth colony and a translucent and flat colony, which was later confirmed as lactose negative variants (Supplementary Figure S1). Adaptation was started with a lactose positive colony but it seems that during this step, only lactose negative variants were selected. In fact, lack of lactose fermentation and loss of a large plasmid band in L98-C1 and L98-E2 was confirmed by the absence of growth in BCP-lac (Supplementary Figure S2C) and plasmid isolation, respectively (Supplementary Figure S2A). Another mutant that lost one plasmid was L62-C9 (Supplementary Figure S2) but in this case, lactose fermentation was not impaired (data not shown).

### Technological Aptitude of Lcn972R Mutants in Milk

The first step in the characterization of the Lcn972R mutants selected after AE-CES was to assess whether dairy technological traits were retained or not. Accordingly, their ability to grow in pasteurized milk was compared to that of their WT counterparts. Similarly, nisin production was examined as well as bacteriophage resistance.

#### Growth and Lactic Acid Production in Milk

**FIGURE 2:**
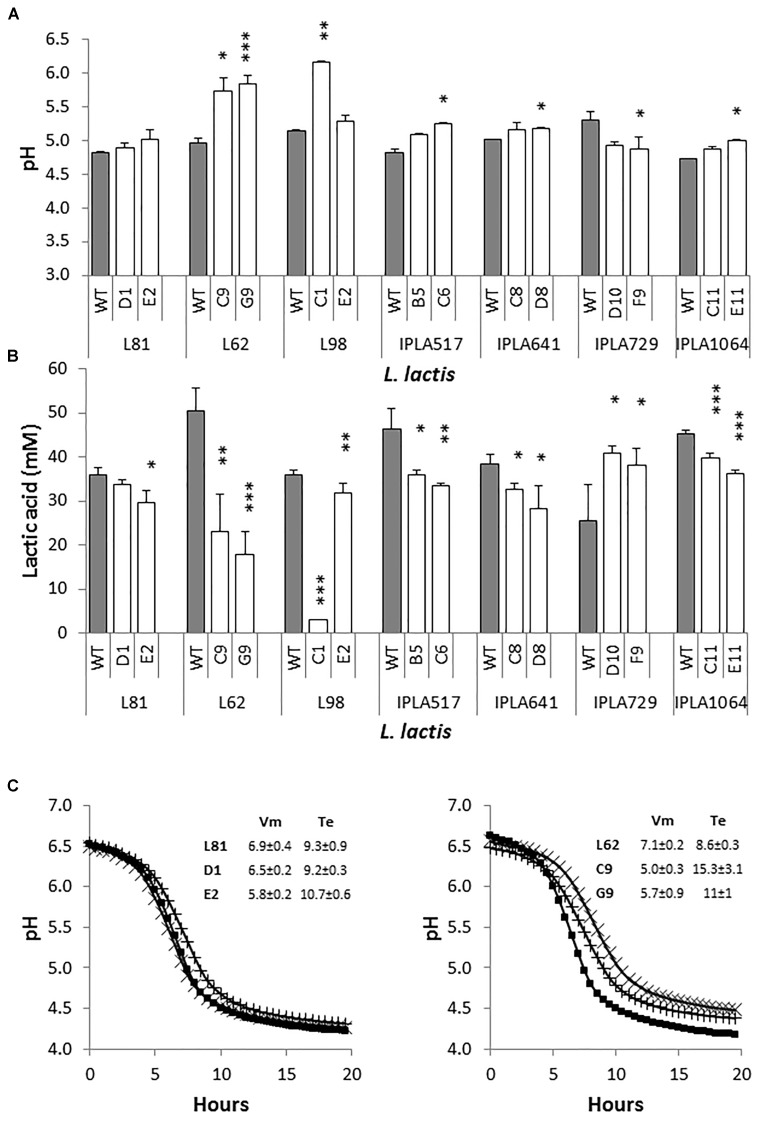
Growth of *L. lactis* in pasteurized milk. pH **(A)** and production of lactic acid **(B)** of *L. lactis* wild-type (gray bars) and their Lcn972R mutants (white bars). Cultures were inoculated at 3% and incubated for 6 h at 30°C. **(C)** Acidification curves of *L. lactis* L81 (left panel) and *L. lactis* L62 (right panel) and their Lcn972R mutants in pasteurized milk. (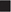), wild type strains; (X), L81-D1 and L62-C9; (+), L81-E2 and L62-G9. The insert shows the acidification curve descriptors. Vm, maximum acidification rate (dpH/dt, mpH/min); Te, time to reach pH 4.6 (h). ^∗^*p* < 0.05, ^∗∗^*p* < 0.01, ^∗∗∗^*p* < 0.001 significantly different to the wild type strain.

Starting with overnight cultures in LM17, pasteurized milk was inoculated at 3% (v/v) and pH as well as the production of lactic acid of milk cultures was measured after 6 h of incubation at 30°C (Figure 2). All strains acidified to a pH below a threshold of 5.3, regarded as a standard for a dairy starter under these experimental conditions (Cogan et al., 1997). The exception was the Lcn972R mutants of the starter *L. lactis* L62 and the lactose negative *L. lactis* L98-C1 which hardly acidified milk. In addition, the mutant L98-E2, that was shown to be also lactose negative in broth, lowered the pH as much as the WT *L. lactis* L98 (*p* > 0.05) (Figure 2A). This behavior may reflect the ability of this mutant to use other energy sources from milk.

Differences were observed in the production of lactic acid after 6 h of incubation (Figure 2B). In general, the amount of lactic acid generated by the Lcn972R mutants ranged from 70 to 90% of that produced by their parental strains with the exception of *L. lactis* IPLA729-D10 and F9 with higher levels compared to the WT IPLA729. *L. lactis* L62-C9 and L62-G9 produced less than 50% of the lactic acid levels of their WT strain. These results suggested a long lag phase in milk when these mutants were transferred from LM17 broth to milk and/or a slower acidification rate. To get a better picture of the acidification behavior of these mutants in milk, acidification was followed measuring the pH with a real-time pH meter for 20 h. As a reference, acidification curves for *L. lactis* L81 and its Lcn972R mutants were also performed (Figure 2C). In agreement with the pH and lactic acid concentration determined after 6 h of incubation (Figures 2A,B), *L. lactis* L81-D1 behaved as its WT, while L81-E2 was slightly slower (*p* < 0.05) in terms of maximum acidification rate (Vm) (Figure 2C). Similarly, the mutants L62-C9 and L62-G9 showed a slower maximum acidification rate (*p* < 0.05) and it took 7 and 2.5 h longer (Te), respectively, to reach pH 4.6 compared to *L. lactis* L62 (Figure 2C). Therefore, these two Lcn972R mutants had difficulties to grow in milk, were slower in lowering the pH and were not able to reach the same pH as *L. lactis* L62.

#### Nisin Production

**FIGURE 3:**
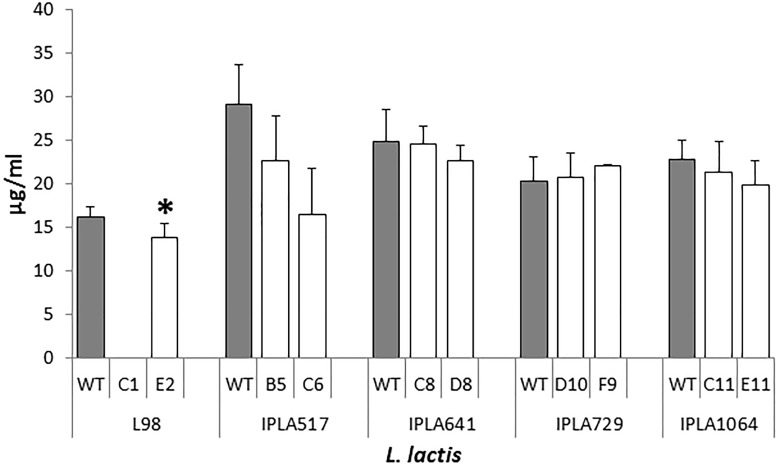
Nisin production by *L. lactis* and their Lcn972R mutants (white bars) in pasteurized milk incubated at 30°C for 6 h. ^∗^, significantly different (*p* < 0.05) from the wild-type (gray bars).

All the nisin producers were able to synthesize nisin in LM17 broth (data not shown), but the production of nisin in milk was investigated as a key technological trait which should be retained by the evolved strains. As shown in Figure 3, no significant differences (*p* > 0.05) were observed with the Lcn972R mutants derived from the nisin Z producing strains isolated from raw milk cheeses. As expected, no detectable levels of nisin were measured in L98-C1 milk cultures, due to the absence of growth. On the contrary, the mutant L98-E2 did produce nisin, despite its lactose negative phenotype, although levels were slightly lower (*p* < 0.05) than the WT L98 (Figure 3).

#### Bacteriophage Resistance

**FIGURE 4:**
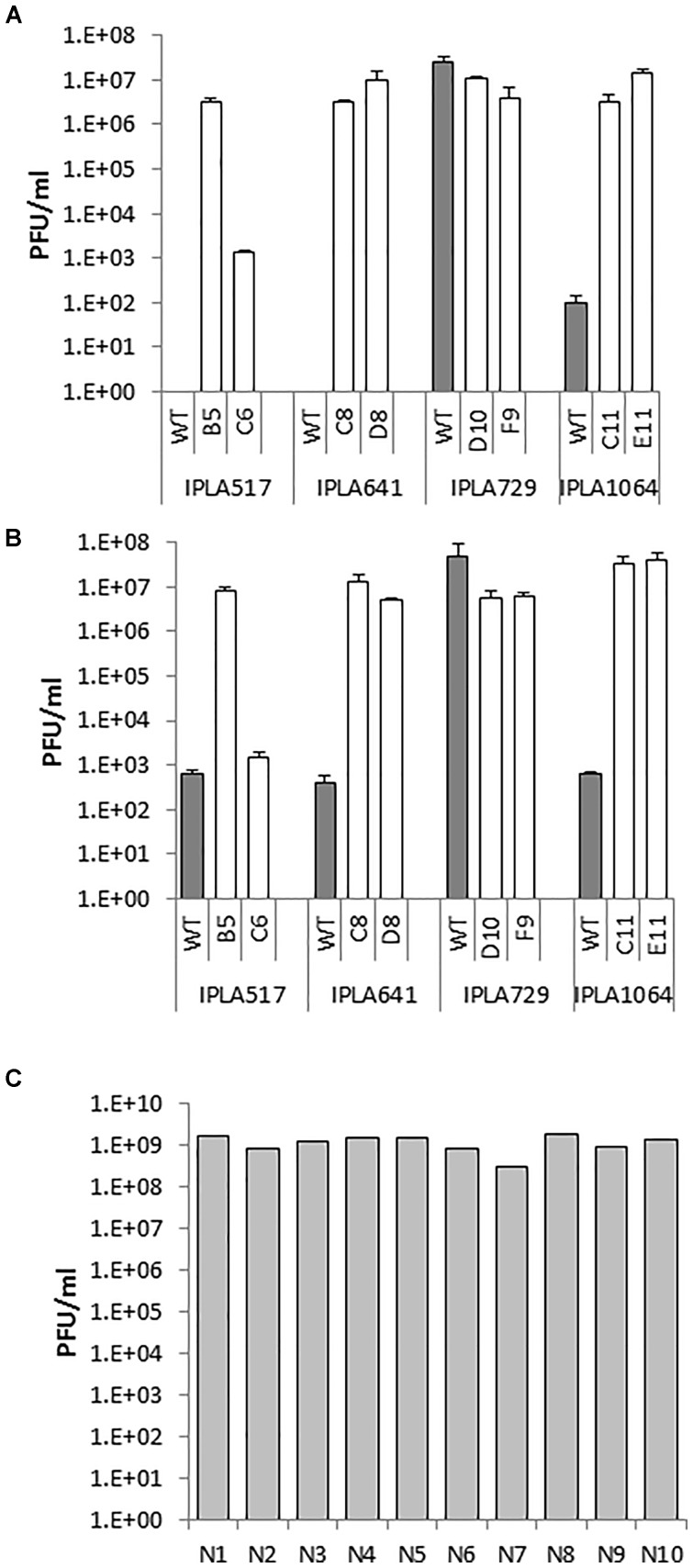
Phage susceptibility of *L. lactis* IPLA517, IPLA641, IPLA729, and IPLA1064 (gray bars) and their Lcn972R mutants (white bars). Phage titer was determined by the double layer assay. **(A)** Phage CHPC1130 (1.5 × 10^10^ PFU/ml). **(B)** Phage CHPC1180 (4.6 × 10^9^ PFU/ml). **(C)** titers of phage CHPC1130 on novobiocin plasmid-cured derivatives (N1–N10) of *L. lactis* IPLA517.

Another technological trait of interest is bacteriophage resistance since bacteriophages are one of the leading causes of fermentation failures worldwide (Garneau and Moineau, 2011). Thus, the susceptibility of the Lcn972R mutants to phages infecting the WT strains was examined. Phages infecting the industrial strains L81, L62, and L98 still propagate and reach similar phage titers when plated on the Lcn972R mutants (data not shown). Since the phage susceptibility profile of the nisin Z producers was unknown, a growth inhibition assay was initially carried out to select phages able to infect the WT strains. *L. lactis* IPLA517, IPLA641, IPLA729, and IPLA1064 were screened against 62 lactococcal phages. Five phages, all belonging to the c2 family, strongly impaired acidification of all strains. Additionally, *L. lactis* IPLA729 was also inhibited by two extra c2 phages and *L. lactis* IPLA641 was partially inhibited by a 936 family phage and by an unclassified one (Supplementary Table S1). Two c2 phages, CHPC1130 and CHPC1183, were chosen to study further their infectivity on the Lcn972R mutants. Despite the effect observed in the growth inhibition assay, none or just a few lysis plaques were detected when plating undiluted lysates of CHPC1130 (1.5 × 10^10^ PFU/ml) and CHPC1183 (4.6 × 10^9^ PFU/ml) onto the WT strains IPLA517, IPLA641, and IPLA1064 (Figure 4). On the contrary, the phages did plaque on their Lcn972R mutants and lysis plaques were observed with titers up to 10^7^ PFU/ml (Figure 4). No changes in phage susceptibility were detected for *L. lactis* IPLA729 and its Lcn972R mutants.

We attempted to find a plausible explanation for the phage infectivity in the Lcn972R mutants. Since plasmid loss had been already observed for some Lcn972R mutants, we speculated that a putative plasmid-encoded phage resistance mechanism could have been lost during adaptation. To test this hypothesis, we attempted to compare the plasmid profiles of these strains and their Lcn972R mutants but it was not possible to clearly discriminate if any of the plasmids were lost (data not shown). As an alternative, we used novobiocin as a plasmid-curing agent to cure plasmids from *L. lactis* IPLA517. After several passages, ten single colonies were grown and tested against the phage CHPC1130. Lysis plaques were observed in all of them with titers even higher than those found on the Lcn972R mutant 517-B5 (Figure 4C). All the cured clones seem to have lost several large plasmids present in *L. lactis* IPLA517 (Supplementary Figure S3). Based on these results, a likely explanation for the phage susceptibility of the Lcn972R mutants is the loss of plasmid-encoded anti-phage mechanisms.

### Surface Hydrophobicity and Autolytic Behavior of Lcn972R Mutants

A common theme in bacteriocin resistance is the existence of changes of the physicochemical properties of the bacterial surface to prevent or reduce binding of the antimicrobial peptide to the cell (Kramer et al., 2008; Bastos et al., 2015). Moreover, alterations on the bacterial surface may influence the activity of autolysins (Steen et al., 2005). In this context, we studied cell hydrophobicity and autolysis within the Lcn972R mutants. In these experiments, the starter *L. lactis* IPLA947 and its Lcn972 resistant derivative *L. lactis* R5 were also included.

**FIGURE 5:**
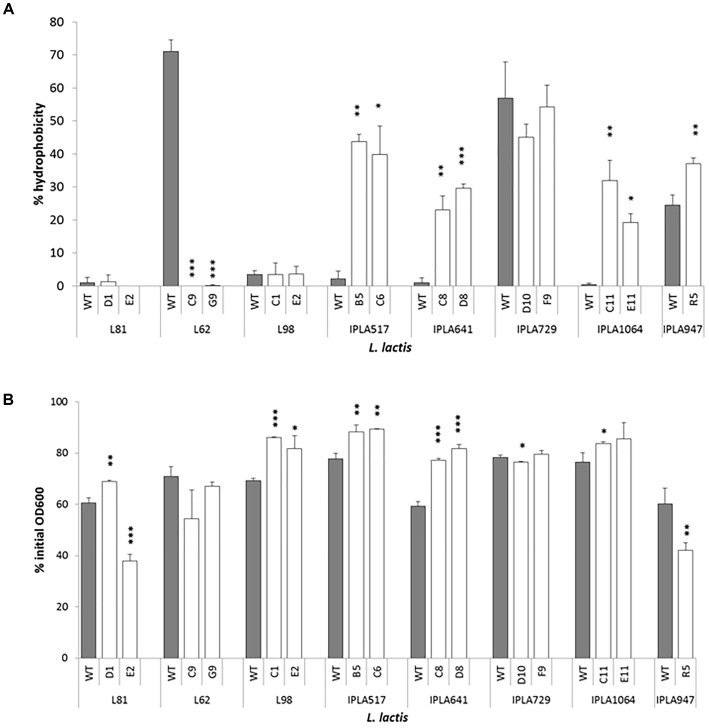
Surface hydrophobicity **(A)** and autolysis **(B)** of *L. lactis* strains and their Lcn972R mutants. For autolysis, the OD_600_ was measured after 150 min of incubation of cell suspensions at 30°C. ^∗^*p* < 0.05, ^∗∗^*p* < 0.01, ^∗∗∗^*p* < 0.001 significantly different to the wild type strain.

Nine Lcn972R mutants changed their surface hydrophobicity with respect to the parental strains (Figure 5A). Within the group of nisin Z producers IPLA517, IPLA641, and IPLA1064 and the starter IPLA947, their Lcn972R mutants were more hydrophobic with values between 20 and 40%, as measured by their transfer to the hexadecane phase in the MATS test. A dramatic shift in surface hydrophobicity was noted for L62 whose Lcn972R mutants completely lost its hydrophobic character. Mutants from *L. lactis* L81, L98, and IPLA729 did not suffer any major changes in surface hydrophobicity.

As to their autolytic behavior, the Lcn972R mutants became less autolytic than their parental strains (Figure 5B) with two exceptions: *L. lactis* L81-E2 and *L. lactis* R5. In both Lcn972R mutants, down to 40% of the initial OD_600_ was recorded after 150 min in the presence of triton X-100 while 60% was recorded for the WT strains. The Lcn972R mutants from *L. lactis* L62 behave as the WT strain (Figure 5B). It is interesting to note that the two Lcn972R mutants from the same parental strain, e.g., *L. lactis* L81, behaved opposite to each other with regard to their autolytic activity (Figure 5B).

### Cross-Resistance to Technological Stresses and Cell Wall Antimicrobials

Considering that Lcn972 inhibits cell wall biosynthesis and triggers the cell envelope stress response in *L. lactis*, it was anticipated that mutations leading to changes in cell wall structure or composition could have been selected for during adaptation. Because the cell wall is crucial for survival (Chapot-Chartier and Kulakauskas, 2014), we presumed that these possible alterations could make Lcn972R mutants to withstand better other stresses such as heat, low pH, and high osmotic pressure, all of them technological stresses encountered during cheese production. Besides, cross-resistance to other antimicrobials acting at the cell wall might arise, as previously observed with the laboratory strain *L. lactis* MG1614 that became resistant to lysozyme and nisin (Roces et al., 2012a). Therefore, the following experiments were designed to compare the response of the Lcn972R mutants to their parental strains.

#### Cross-Resistance to Heat and Low pH

**FIGURE 6:**
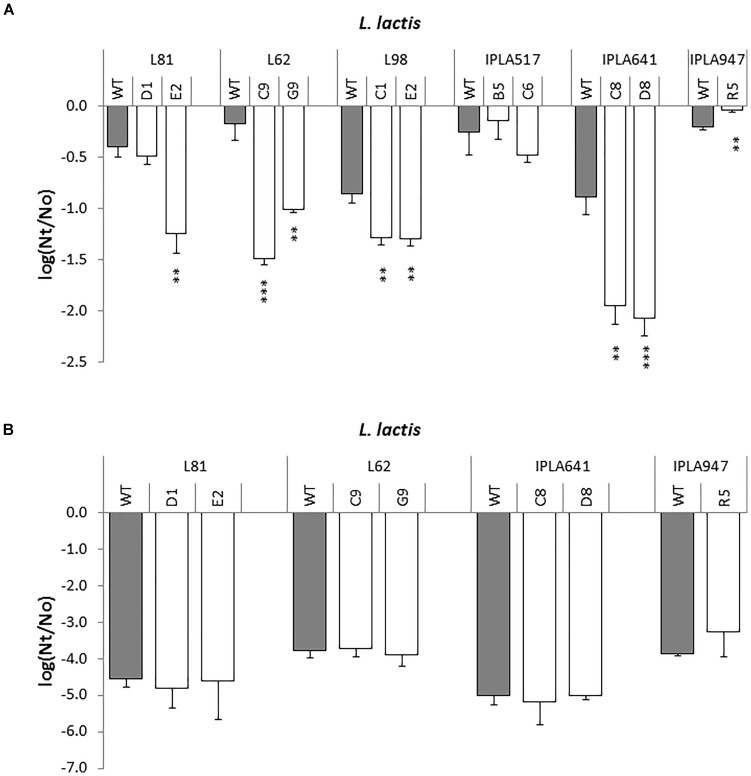
Survival of *L. lactis* (gray bars) and their Lcn972R mutants (white bars) after exposure to heat **(A)** and low pH **(B)**. ^∗^*p* < 0.05, ^∗∗^*p* < 0.01, ^∗∗∗^*p* < 0.001 significantly different to the wild type strain.

A representative set of Lcn972 mutants, including those from the industrial *L. lactis* L81, L62, and L98, two nisin Z producers IPLA517 and IPLA641, and the dairy starter IPLA947 were exposed to 50°C and the viability was determined after 30 min (Figure 6A). Out of the eleven Lcn972R mutants, one, *L. lactis* R5, was more resistant to heat shock and three equally resistant to their wild types (L81-D1, IPLA517-B5, and IPLA517-C6). All the others became sensitized and lost around 1 log unit more than their wild types (Figure 6A). Once again, the two Lcn972R mutants from L81 and L62 behaved opposite to each other. On the contrary, none of the Lcn972R mutants became more sensitive to low pH. For all the strains tested, exposure to pH 2 led to a 4–4.5 log units decrease in viability (Figure 6B) and no significant differences (*p* > 0.05) were observed for any of the Lcn972R mutants and their parental strains.

#### Cross-Resistance to NaCl and Cell Wall Antimicrobials

Cross resistance was qualitatively evaluated by spotting decimal dilutions of exponentially growing cells on LM17 plates, supplemented with bacitracin, lysozyme, penicillin G and vancomycin at the concentrations indicated in Table 2. Growth under hyperosmotic conditions was assessed on LM17 plus NaCl at 5% (0.86 M). Resistance to nisin was only examined for non-nisin producers. As a general trend, half of the Lcn972R mutants became sensitive to penicillin G (60%) and to NaCl (46%). On the contrary, most of them (73%) were more resistant to lysozyme and to vancomycin (50%), while cross-resistance to bacitracin was observed in four cases only. Among the non-nisin producers, three out of five Lcn972R mutants showed cross-resistance to nisin. Taken as a whole, Lcn972R mutants from the same strain did not phenocopy each other and only those derived from *L. lactis* L98 and IPLA641 exhibited exactly the same phenotypes (Table 2).

**Table 2:** Cross-resistance to cell wall antimicrobials and NaCl of *L. lactis* mutants resistant to Lcn972.

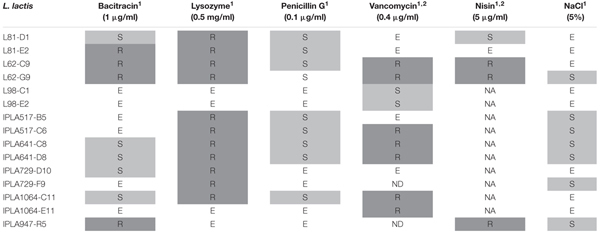

*^*1*^S, E and R stand for more susceptible, equal and more resistant than the wild type counterpart. Susceptibility/resistance was determined by spotting decimal dilutions of cell suspensions on LM17 plates supplemented with the indicated concentrations.*

*^*2*^NA, not applicable; ND, not determined.*

### Overview of Non-synonymous Mutations Within Lcn972R Mutants

**FIGURE 7:**
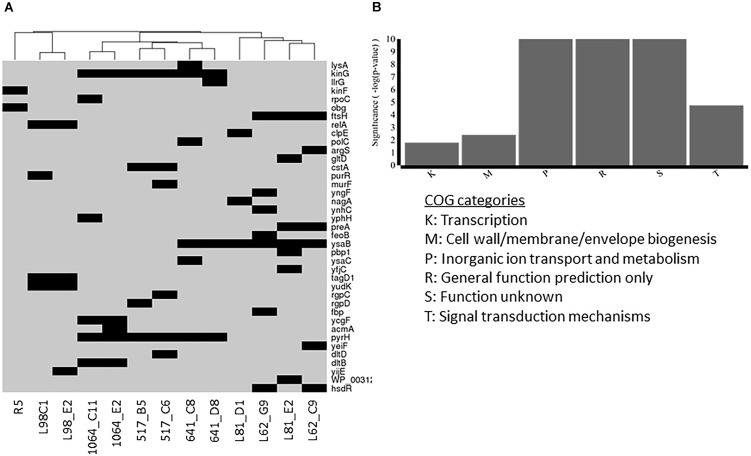
Non-synonymous mutations present in Lcn972R *L. lactis* mutants **(A)** and functional categories overrepresented within the mutated genes **(B)**. Presence of mutations in **(A)** is represented by a black rectangle.

To gain a preliminary insight into the genetic diversity introduced during evolution in the presence of Lcn972 and identify single mutations (and possible compensatory-mutations) selected during the adaptation-stabilization steps, draft genome sequences of the Lcn972R mutants from *L. lactis* L81, L62, L98, IPLA517, IPLA641, IPLA 1064 as well as the Lcn972R mutant from IPL947, *L. lactis* R5, were analyzed (Figure 7 and (Supplementary Table S2).

Two to seven non-synonymous mutations were detected in the Lcn972R mutants within a total of 39 genes. Among them, 23 were predicted to affect protein function according to the SIFT algorithm or a frameshift and/or a stop codon was introduced (Supplementary Table S2). Noticeably, mutants clustered together according to the parental strain. Lcn972R *L. lactis* from the protective culture L98 and R5 displayed an independent set of mutated genes, while nisin Z producers clustered together and apart from the commercial starters *L. lactis* L81 and L62 (Figure 7A). Taken together, these results reinforce the notion that AE-CES may lead to different outputs depending of the genetic background of the parental strain.

Six main COGs categories were overrepresented in the gene set were non-synonymous mutations were detected (Figure 7B). Not surprisingly, some mutations were found in genes involved in cell envelope biogenesis (COG M) such as *murF* (D-Ala-D-Ala adding enzyme), *tagD1* (glycerol-3-phosphate cytidylyltransferase) and *pbp1* (penicillin binding protein 1A/1B) detected in IPLA517-C6, L98-C1 and E2, and L81-E2, respectively. Two genes *dltB* and *dltC* encoding functions involved in D-alanylation of the lipoteichoic acid (LTA) were also mutated in *L. lactis* IPLA1064-C11 and E11 and IPLA517-C6, respectively. Mutations in the genes *rgpCD*, coding for a putative ATP binding cassette (ABC) transporter likely involved in the synthesis of the polysaccharide pellicle (PSP), were also detected in the two Lcn972R mutants from *L. lactis* IPLA517.

Mutations in signal transduction mechanisms and transcriptional regulators were also found in several strains, anticipating a complex scenario at the transcriptional, and thus, phenotypic level. Namely, the two component system TCS-G accumulated mutations in the six Lcn972R mutants derived from the nisin Z producers. Other four mutants from the starters *L. lactis* L81 and L62 revealed mutations in *ysaBC* coding for a BceAB-like ABC transporter adjacent to TCS-G. Therefore, the most frequent mutations among Lcn972R mutants were found in the BceAB-like TCS module which in a model organism such as *Bacillus subtilis* is involved in sensing and resistance to antimicrobial peptides (Ohki et al., 2003). Mutations in other stress-related genes were also found in *ftsH*, coding for the membrane protease FtsH involved in protein quality control and regulatory functions, and in *relA*, encoding the alarmone ppGpp synthetase. Nucleotide metabolism appeared to be also affected in some Lcn972R mutants. The uridylate kinase gene involved in pyrimidine metabolism (*pyrH*) was mutated in all the Lcn972R mutants from IPLA517, IPLA641, and IPLA1064 as well as the purine operon regulator *purR* in *L.lactis* L98-C1.

## Discussion

Lactic acid bacteria bacteriocins have been traditionally recognized as potent antimicrobials to be employed in food biopreservation. However, narrow-spectrum bacteriocins targeting beneficial bacteria (e.g., the dairy starter *L. lactis*) also have a niche of application in food biotechnology. For example, premature lysis of starter cells induced by the anti-lactococcal bacteriocins lactococcin A, B, and M facilitated the release of intracellular enzymes and accelerated cheese ripening (Morgan et al., 1997). In this work, we have made use of the narrow spectrum bacteriocin Lcn972 in evolution experiments with the aim of introducing diversity within *L. lactis* industrial starters. Our hypothesis was supported by our previous results on the characterization of Lcn972R mutants from the laboratory strain *L. lactis* MG1614 and the starter *L. lactis* IPLA947 but, in a way, this work was also inspired by the wealth of knowledge gained in the antibiotic field that clearly verified how low-level drug exposure generates genetic and phenotypic variability within pathogenic bacteria (Andersson and Hughes, 2014). On the other hand, evolution experiments have already been undertaken in *L. lactis* (see Bachmann et al., 2017 and references therein). These studies have demonstrated the genomic plasticity of *L. lactis* to fix beneficial mutations when exposed to particular conditions. However, most of these studies have been carried out as proof of concept studies, using laboratory strains that differ greatly from industrial or natural *L. lactis* strains (Cavanagh et al., 2015; Kelleher et al., 2017).

On this basis, we have applied AE-CES to seven *L. lactis* strains, including commercial and isolates from raw milk cheeses, to gather information about the influence of the strain background. It was possible to select Lcn972R mutants in all cases, supporting the idea that using Lcn972 in AE-CES experiments could be extended to virtually all Lcn972-sensitive *lactococci*. Moreover, in contrast to nisin resistant *Lactococcus*, that required the presence of nisin to retain their resistant phenotype (Kramer et al., 2006), Lcn972R mutants were stable, growth under laboratory conditions was not impaired and their resistance was not lost upon subcultivation. Two factors might have contributed to their stability. One is a possible high mutation rate in cells exposed to Lcn972. Lcn972 inhibits cell wall biosynthesis during cell division without pore formation (Martínez et al., 2008). So, cells are not instantly killed, increasing the chance to select mutations. Besides, it is known for several antibiotics, including cell wall antibiotics that mutation frequency increases when the SOS response is activated (Gutierrez et al., 2013). Although it has not been experimentally confirmed, Lcn972 seems to trigger the SOS response, as evidenced by the activation of prophages after Lcn972 treatment (Madera et al., 2009). A second factor that may have influenced the selection for stable mutants is the stabilization step after Lcn972 adaptation. Competition between clonal variants may have helped fixing beneficial compensatory mutations and the selection for fitter variants.

Aside from the physiological implications of using Lcn972 as a stressor in AE-CES, a key question was if this strategy would be compatible with the functionality of the dairy starters. The first issue we addressed was the technological characterization of the Lcn972R mutants. In general, milk acidification was within the standards for dairy starters (Cogan et al., 1997) and, half of the mutants retained the same acidification rate as their corresponding WT. On the other hand, lactic acid levels were in most cases lower, suggesting a shift from homolactic to a more mixed-acid fermentation as a possible consequence of the adaptation to Lcn972. Whether this shift results in higher acetaldehyde or diacetyl levels or not remains to be investigated. In addition, nisin production levels were not altered with the exception of the lactose negative mutants L98-C1 and E2. Taken together, the results showed that evolved mutants that retained the wild type technological phenotypes may be recovered after AE-CES.

However, a major disadvantage encountered during AE-CES was the loss of plasmids in some strains. The plasmid complement of *Lactococci* is large and several relevant technological traits are plasmid-encoded, from lactose fermentation, proteolytic activity to bacteriophage resistance mechanisms (Ainsworth et al., 2014b). Two examples of the negative consequences of plasmid loss have been observed in this work. One was the loss of the lactose plasmid in the Lcn972R mutants from *L. lactis* L98 that occurred in spite of the presence of lactose as a carbon source during AE-CES. In the wild type *L. lactis* L98, the lactose plasmid appears to be intrinsically unstable, as described for other lactose plasmids such as pLP712 (Wegmann et al., 2012), and only lactose negative variants were selected during adaptation. The other example was the loss of a plasmid (or plasmids) encoding phage resistance mechanisms that occurred in *L. lactis* IPLA517 and, most likely, in the other nisin Z producing strains IPLA641 and IPLA1064. Presence of plasmid-encoded anti-phage mechanisms in the wild type strains is supported by the isolation of phage sensitive clones after plasmid curing using novobiocin, although chromosomal re-organization events might have occurred as well. Two scenarios may take place. A putative plasmid-encoded exopolysaccharide (EPS) could shield the phage receptor, as described for the lactococcal plasmid pCI658 that encodes the production of an EPS protecting *Lactococcus* from infection by phages phi712 and c2 (Forde and Fitzgerald, 2003). In our case, differences in the expression level of the EPS genes in LM17 and in milk may explain why no lytic plaques were observed when phages were plated on the WT strains, despite the inhibition observed in the growth inhibition test. Alternatively, loss of genes coding for abortive infection mechanisms (Abi) may also explain why Lcn972R mutants became phage sensitive and the lack of lysis plaques on otherwise “phage sensitive” *L. lactis*. Abi systems are altruistic phage resistant mechanisms, whereby the phage cycle is stopped and infected cells die before the phage ends its intracellular cycle (Labrie et al., 2010). Thus, progeny phages are not released, i.e., lysis plaques are not observed, and milk acidification is slowed down due to cell death. So far, searching draft genomes for putative Abi systems retrieved no relevant hits. On the contrary, a putative EPS gene cluster flanked by insertion sequences seems to be present in *L. lactis* IPLA517, IPLA641, and IPLA1064 and absent in their Lcn972R mutants and in the phage sensitive *L. lactis* IPLA729. Unfortunately, the identified contigs basically contain genes involved in EPS synthesis and the absence of any plasmid-related sequences next to the EPS genes precludes us from confirming the plasmidic nature of the EPS operon. Nevertheless, reads matching plasmid replication genes were also missing in the genomes of the Lcn972R mutants, further confirming plasmid loss in the mutants as already observed in plasmid preparations. Based on these examples, and considering that both lactose fermentation and phage resistance are key technological dairy traits, caution must be taken when applying AE-CES depending on the strains and their plasmid complement.

Further phenotypic characterization of the Lcn972R mutants, complemented by the preliminary overview of non-synonymous mutations, supports one of the outcomes of this study. That is both the inter- and intra-strain variability observed among the evolved mutants. In other words, the results emphasize the different solutions found by *L. lactis* to overcome the stress imposed by Lcn972. In spite of the relative low number of Lcn972R mutants characterized per strain (*n* = 2), different phenotypes were observed. This is the case, for instance, of the two *L. lactis* L81 Lcn972R mutants that behaved opposite to each other regarding autolysis and heat susceptibility. Moreover, as discussed below, the phenotypes selected after AE-CES also differed from those described for the laboratory strain *L. lactis* MG1614 (Roces et al., 2012a,b).

Changes that happened at the cell surface are exemplified by alterations on surface hydrophobicity and the autolytic behavior of the Lcn972R. Seven mutants increased their surface hydrophobicity, whereas L62-C9 and G9 lost completely the hydrophobic character of their WT. Interesting, the phage sensitive Lcn972R mutants derived from IPLA517, IPLA641, and IPLA1064 became hydrophobic, likely due to the loss of a putative hydrophilic EPS involved in phage resistance (see above). Autolysis was more uniform within Lcn972R mutants and, in general, evolved strains were less autolytic than the WT reference. These new surface properties may have a technological impact as well. It has been recently shown that the properties of *L. lactis* cell surface affects textural parameters of fermented milk (Tarazanova et al., 2018). Moreover, autolytic *L. lactis* may influence the development of cheese flavor (Lortal and Chapot-Chartier, 2005), opening an avenue for new applications of some of the evolved starters such as *L. lactis* L81-E2.

Considering that the cell wall is crucial for survival, one of our initial premises based on the mode of action of Lcn972 was that mutations leading to resistance to Lcn972 could have positive consequences on the survival to other stresses encountered during cheese manufacture. For example, extensive remodeling of the cell wall by increasing D-Asp amidation, *O*-acetylation, and *N*-deacetylation of the peptidoglycan promoted acid resistance in *L. lactis* (Hao et al., 2017; Cao et al., 2018). However, cross-protection to low pH was not observed within Lcn972R mutants. Furthermore, resistance to Lcn972 seems to be linked to a higher susceptibility to heat and NaCl (see Figure 6 and Table 2). Therefore, AE-CES using Lcn972 does not necessarily evolve robust *L. lactis*, at least, for this group of strains and the studied phenotypes. In contrast, cross-resistance to other cell wall antimicrobials was found. Increased lysozyme tolerance and cross-protection to other bacteriocins appears to be a common theme among bacteriocin resistance mutants (see Bastos et al., 2015 and references therein), regardless the mode of action, i.e., pore-forming or cell wall inhibiting bacteriocins. Likewise, sensitivity to β-lactam antibiotics is often observed (Guinane et al., 2006; Roces et al., 2012a) and our results are in line with these reports.

The preliminary overview on the non-synonymous mutations detected in the Lcn972R mutants revealed, first of all, that selected mutations seem to be dictated by the background of the parental strain. Yet, distinct intra-strain mutation profiles were observed in agreement with the notion that “in an evolutionary trajectory, multiple solutions may lead to a fitness increase” (Bachmann et al., 2017). These diversity may have been introduced in both adaptation and stabilization steps.

Mutations were preferentially found in genes involved in cell envelope biogenesis, active transport and regulatory functions. It should be noted that mutations that are predicted to affect protein function do not necessarily mean loss of function. Thus, the interpretation of the impact of a given mutation should be taken cautiously at this stage. With this in mind, it is fair to assume that the mutations found in the BceAB-like *ysaCB*-TCS-G module, the most frequent within Lcn972R mutants, may lead to activation of this detoxification module rather than to its inactivation and, thereby, provide resistance against bacitracin, vancomycin and Lcn972 as well. Mutations in the ABC permease component in *Streptococcus pneumoniae* led to increased transcription of the ABC transporter genes and resistance to vancoresmycin (Becker et al., 2009). Further studies are in progress to confirm if the same applies to our Lcn972R mutants.

Another frequent mutation was found in *pyrH*, encoding the UMP kinase involved in the last steps of the synthesis of RNA precursors. This mutation may have direct consequences on the architecture of the cell wall and turn cells resistant to cell wall antimicrobials as lysozyme and Lcn972. Recently, mutations in *pyrB*, coding for the aspartate carbamoyltransferase in pyrimidine metabolism, were frequently found in *L. lactis* resistance to lysozyme (Solopova et al., 2016). The authors claimed that limiting the availability of L-Asp for nucleotide biosynthesis results in a highly cross-linked and rigid peptidoglycan that interferes with lysozyme activity. Other mutations that may have an impact on surface properties are those found in the mutants from IPLA517 in the genes *rgpCD* located in the cell wall polysaccharide gene cluster. These genes putatively encoded the ABC transporter of the polysaccharide precursors and, when non-functional, the amount of the polysaccharide pellicle may be reduced, increasing surface hydrophobicity. This cell wall polysaccharide is a well-stablished receptor for several *L. lactis* phages of the 936 and P335 groups (Ainsworth et al., 2014a). Unfortunately, none of the phages able to infect the WT *L. lactis* IPLA517 belong to any of these phage groups and it could not be established if these mutations may render IPLA517 less prone to phage attack. Finally, absence of growth in milk of *L. lactis* L98-C1 could be linked to the mutation in *purR*. The inactivation of this transcriptional activator would potentially render the mutant unable of *de novo* synthesis of purines required for growth in milk, a substrate low in purine content (Kilstrup and Martinussen, 1998). Interestingly, mutations connected to purine nucleotide metabolism are often behind multi-stress resistance in *L. lactis* (Ryssel et al., 2014) which may explain the selection of such mutations during AE-CES.

## Conclusion

In this work we have identified advantages and disadvantages of applying AE-CES in *L. lactis* and the consequences for its performance as a dairy starter. AE-CES appears as a feasible strategy to introduce phenotypic and genetic diversity in *L. lactis*, regardless of the strain origin. Evolved strains may retain similar technological traits as the strains they are derived from, while acquiring new ones. Plasmid loss was one of the main disadvantages that might be overcome by combining AE-CES with conditions for selection of the plasmid of interest or by screening a larger number of evolved clones. Although still preliminary, data gathered from draft genomes anticipates the likely selection for mutations activating detoxification modules and changes at the cell surface which may have practical implications in milk fermentations. The phenotypic and genetic characterization of the evolved strains has also emphasized the plasticity of *L. lactis* to give rise to new phenotypes, providing the versatility required for adaptive evolution to become an excellent tool in strain development programs.

## Data Availability

Datasets are available on request. The raw data supporting the conclusions of this manuscript will be made available by the authors, without undue reservation, to any qualified researcher.

## Author Contributions

ML-G ran the evolution experiments. ML-G, TJ, and BM performed the phenotypic characterization experiments. SE, TJ, and BM analyzed mutations. AR, AN, TJ, and BM conceived and designed the study. All authors were involved in analysis of the results and drafting the manuscript prior to submission. Tasks were supervised by AR and BM at IPLA-CSIC and by AN at Chr. Hansen A/S.

## Conflict of Interest Statement

AN and TJ are employees of Chr. Hansen A/S. The remaining authors declare that the research was conducted in the absence of any commercial or financial relationships that could be construed as a potential conflict of interest.

## Footnotes

1 https://blast.ncbi.nlm.nih.gov/

2 http://gseapro.molgenrug.nl/

3 http://genome2d.molgenrug.nl/

4 http://sift.bii.a-star.edu.sg/
