# Supplementary material for: Adaptive Evolution of Industrial Lactococcus lactis Under Cell Envelope Stress Provides Phenotypic Diversity
Source: Front Microbiol. 2018 Nov 5;9:2654. doi: 10.3389/fmicb.2018.02654 (PMC6230721; doi:10.3389/fmicb.2018.02654)
Supplement: Supplementary file 1 [file Data_Sheet_1.docx]

Supplementary Material

Adaptive evolution of industrial *Lactococcus lactis* under cell envelope stress provides phenotypic diversity

**María Jesús López-González^a^, Susana Escobedo^a^, Ana Rodríguez^a^, A. Rute Neves^b^, Thomas Janzen^b^, B. Martínez^a*^.**

^a^ DairySafe group. Instituto de Productos Lácteos de Asturias, IPLA-CSIC. Paseo Río Linares s/n. 33300 Villaviciosa, Asturias, Spain.

^b^ Chr Hansen A/S. 10-12 Bøge Allé. DK2970, Hørsholm, Denmark.

***Correspondence**:

Beatriz Martínez

bmf1@ipla.csic.es

## Supplementary Figures


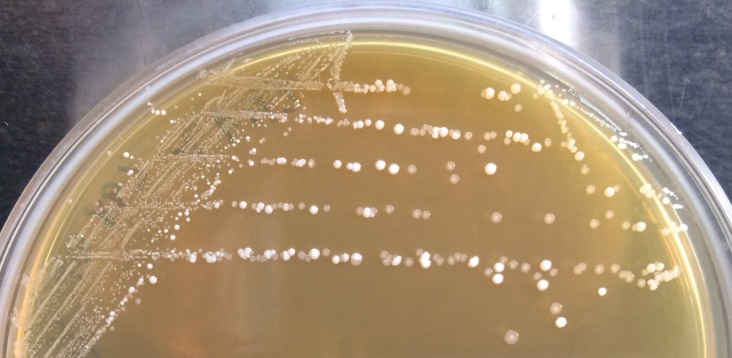


**Supplementary Figure 1.** Colony morphology of *L. lactis* L98 streaked on a LM17 agar plate.


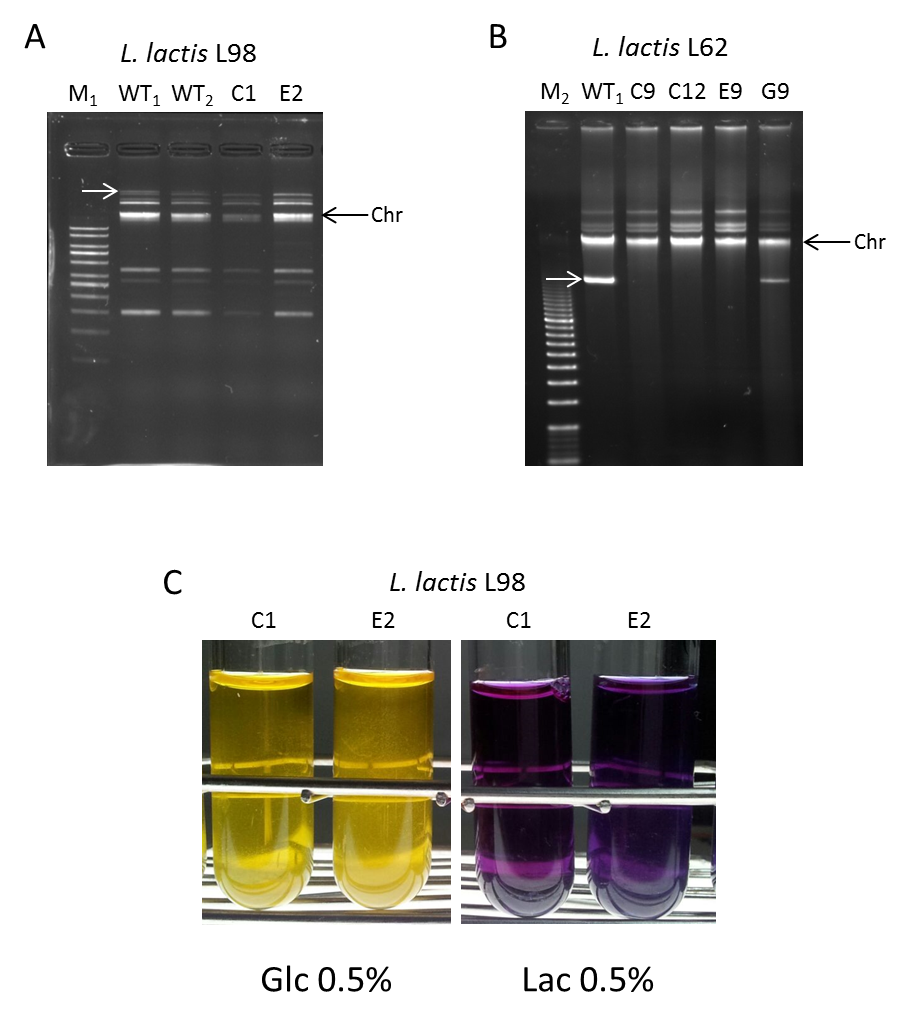


**Supplementary Figure 2.** Plasmid profile of (A) *L. lactis* L98 (WT) and its Lcn972R mutants L98-C1 and -E2 and (B) *L. lactis* L62 (WT) and its Lcn972R mutants L62-C9, -C12,-E9 and -G9. Chr: Chromosomal DNA. M_1_: DirectLoad 1 kb ladder (SIGMA). M_2_: 0.5 kbp ladder (Bio-Rad). The white arrow points to the missing band. (C): Growth and acidification of *L. lactis* L98-C1 and L98-E2 in the basal broth BCP supplemented with glucose or lactose at 0.5%.


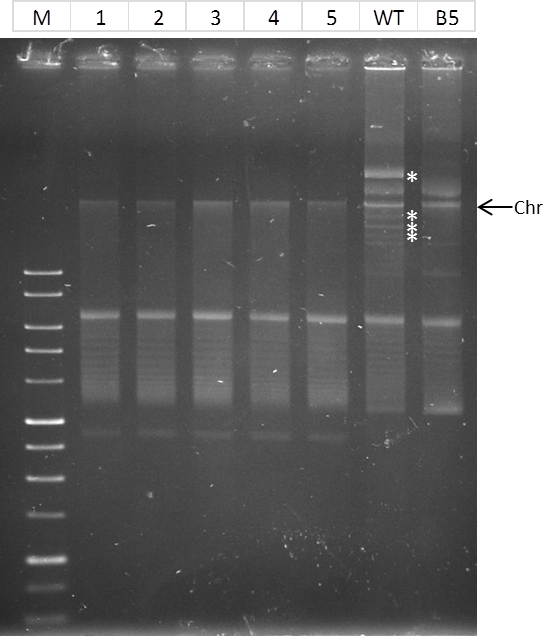


**Supplementary Figure 3.** Plasmid profile of *L. lactis* IPLA517 (WT), its Lcn972R mutant IPLA517-B5 (B5) and five clones (N1 to N5) isolated after novobiocin treatment of *L. lactis* IPLA517. M: 0.5 kb ladder (Bio-Rad). Electrophoresis was carried out in 0.6% agarose gel in TAE buffer at 30 V for 6 h. White starts show plasmidic bands present in IPLA517 and absent in B5.
